# Using CT radiomic features based on machine learning models to subtype adrenal adenoma

**DOI:** 10.1186/s12885-023-10562-6

**Published:** 2023-01-31

**Authors:** Shouliang Qi, Yifan Zuo, Runsheng Chang, Kun Huang, Jing Liu, Zhe Zhang

**Affiliations:** 1 College of Medicine and Biological Information Engineering, Northeastern University, 110169 Shenyang, China; 2 Key Laboratory of Intelligent Computing in Medical Image, Ministry of Education, Northeastern University, 110169 Shenyang, China; 3 Department of Ultrasound Imaging, The First Hospital of China Medical University, 110001 Shenyang, China; 4 Department of Radiology, The First Hospital of China Medical University, 110001 Shenyang, China; 5 Department of Urology, The First Hospital of China Medical University, 110001 Shenyang, China

**Keywords:** Adrenal adenoma, Computed tomography, Radiomics, Machine learning, Radiomic features

## Abstract

**Background:**

Functioning and non-functioning adrenocortical adenoma are two subtypes of benign adrenal adenoma, and their differential diagnosis is crucial. Current diagnostic procedures use an invasive method, adrenal venous sampling, for endocrinologic assessment.

**Methods:**

This study proposes establishing an accurate differential model for subtyping adrenal adenoma using computed tomography (CT) radiomic features and machine learning (ML) methods. Dataset 1 (289 patients with adrenal adenoma) was collected to develop the models, and Dataset 2 (54 patients) was utilized for external validation. Cuboids containing the lesion were cropped from the non-contrast, arterial, and venous phase CT images, and 1,967 features were extracted from each cuboid. Ten discriminative features were selected from each phase or the combined phases. Random forest, support vector machine, logistic regression (LR), Gradient Boosting Machine, and eXtreme Gradient Boosting were used to establish prediction models.

**Results:**

The highest accuracies were 72.7%, 72.7%, and 76.1% in the arterial, venous, and non-contrast phases, respectively, when using radiomic features alone with the ML classifier of LR. When features from the three CT phases were combined, the accuracy of LR reached 83.0%. After adding clinical information, the area under the receiver operating characteristic curve increased for all the machine learning methods except for LR. In Dataset 2, the accuracy of LR was the highest, reaching 77.8%.

**Conclusion:**

The radiomic features of the lesion in three-phase CT images can potentially suggest the functioning or non-functioning nature of adrenal adenoma. The resulting radiomic models can be a non-invasive, low-cost, and rapid method of minimizing unnecessary testing in asymptomatic patients with incidentally discovered adrenal adenoma.

## Introduction

Adrenal adenomas are common tumors, and their prevalence in the general population is about 6% [1], although the incidence increases from about 1% for 40 year-olds to 7% for 70 year-olds [2]. Adrenal adenomas can be divided into functioning adrenocortical adenoma (FAA) and nonfunctioning adrenocortical adenoma (NAA). This classification is mainly based on whether the endocrine function is affected. FAA can disrupt hormone levels in patients, leading to Cushing’s syndrome (CS) and primary hyperaldosteronism (PHA) [3, 4]. Among CS patients, the incidence is high in 20–50 year-olds, and the male-to-female ratio is about 1:3 [5]. For PHA, the incidence is high in 30–50 year-olds, and there is no significant difference in prevalence between men and women [6].

Both FAA and NAA are benign adrenal adenomas. The clinical diagnosis of the two diseases is mainly through endocrine tests for hormone levels or medical imaging [7]. Endocrine analysis requires several blood tests and related hormone induction procedures, which are tedious, time-consuming, and invasive. If NAA is present and there is no hormone abnormality, the hormone test may be unnecessary and delay treatment. Radiologic diagnosis mainly uses morphological information such as the location and size of the lesion. Its accuracy cannot be guaranteed, and misdiagnosis may occur [8], leading to unnecessary adrenalectomy.

Intervention methods are different for the two types of adrenal adenomas. FAA can cause hormone abnormalities, so further examination of hormone levels is required and treatment usually involves surgery [9]. Most NAAs are asymptomatic, and the treatment method is often determined according to the size of the tumor. If the tumor is small and benign, surgical resection is relatively rare [10] and conservative treatment is usually adopted [11], for which no hormone examination is required. Therefore, it is necessary to develop a fast, accurate, and reliable diagnostic method to improve patient intervention and treatment while minimizing unnecessary testing in cases of NAA [12].

Non-invasive computed tomography (CT) can generate high-resolution images and has become a routine examination method. In addition, many features can be extracted from CT images for quantitative analysis [13]. In oncology, texture analysis is a new tool to help diagnose disease [14, 15]. Diagnosis based on machine learning performs as well as experienced doctors in many cases [16, 17]. CT images are assumed to contain valuable information that reflects underlying tumor pathophysiology and quantitative image features (semantic and agnostic), and machine learning models can reveal these relationships [18]. This approach reduces the workload of radiologists and improves the accuracy and efficiency of diagnosis [19–21].

Radiomic studies of adrenal adenomas have been carried out. Elmohr et al. [22] conducted texture analysis on venous phase CT images and established a binary random forest (RF) classification model for benign and malignant large adrenal tumors. Moawad et al. [23] established a random forest classifier to distinguish between benign and malignant uncertain adrenal tumors. Daye et al. [24] used the support vector machine (SVM) classification model to predict the prognosis of ablation patients based on texture features in CT images of adrenal metastases before ablation. In the previous studies of adrenal adenoma, time-consuming manual segmentation of the lesion region was usually required. Moreover, two-dimensional radiomic features were extracted from the largest section, while three-dimensional features that can be extracted from a cuboid containing the adrenal adenoma region were seldom utilized. To the best of our knowledge, differentiation between functioning and non-functioning subtypes of adrenal adenoma using radiomic features and machine learning models has not been reported.

The purpose of this study is to establish an accurate model to distinguish FAA from NAA using CT radiomic features and machine learning methods. This model may provide a non-invasive, low-cost, and rapid method of adrenal adenoma stratification to help patients with NAA avoid unnecessary invasive hormone tests. Our study contributes to this goal in three ways. First, a CT radiomic model is developed for differentiating functioning from non-functioning subtypes of adrenal adenoma in a non-invasive, low-cost, and rapid way. Second, the tedious step of manual segmentation of adrenal adenoma from CT images is replaced by simple cropping of a cuboid containing the target adrenal adenoma region to extract three-dimensional features. Third, a combination of radiomic features obtained from multiple phases of CT images improves classification performance.

## Materials and methods

### Patients and data

Two datasets were collected in this study. Dataset 1 was from The First Affiliated Hospital of China Medical University and was used to develop the machine learning model. Dataset 2 was from Shengjing Hospital of China Medical University and was utilized for external validation. The Ethics Committee of The First Affiliated Hospital of China Medical University and Shengjing Hospital of China Medical University approved this retrospective study, and informed consent was waived.

The experimental subjects were patients who had been diagnosed with adrenal adenomas. The data mainly included patient clinical records and contrast-enhanced CT images. Specifically, the clinical data were the patient’s age, gender, examination time, and pathological type. Each CT scan included images of arterial, venous, and non-contrast phases. The three-phase CT images were used in all cases.

After screening, a total of 289 patients with lesions larger than 2 cm were included in Dataset 1. There were 191 patients with FAA (89 CS, 102 PHA) and 98 with NAA. In subsequent experiments, Dataset 1 was randomly divided into training (n = 201; 133 FAA and 68 NAA) and test (n = 88; 58 FAA and 30 NAA) sets. The training and test sets contained different patients to ensure that they were independent. In Dataset 2, there were 54 patients (30 FAA and 24 NAA). Table 1 summarizes basic patient information and adrenal adenoma characteristics.

**Fig. 1:**
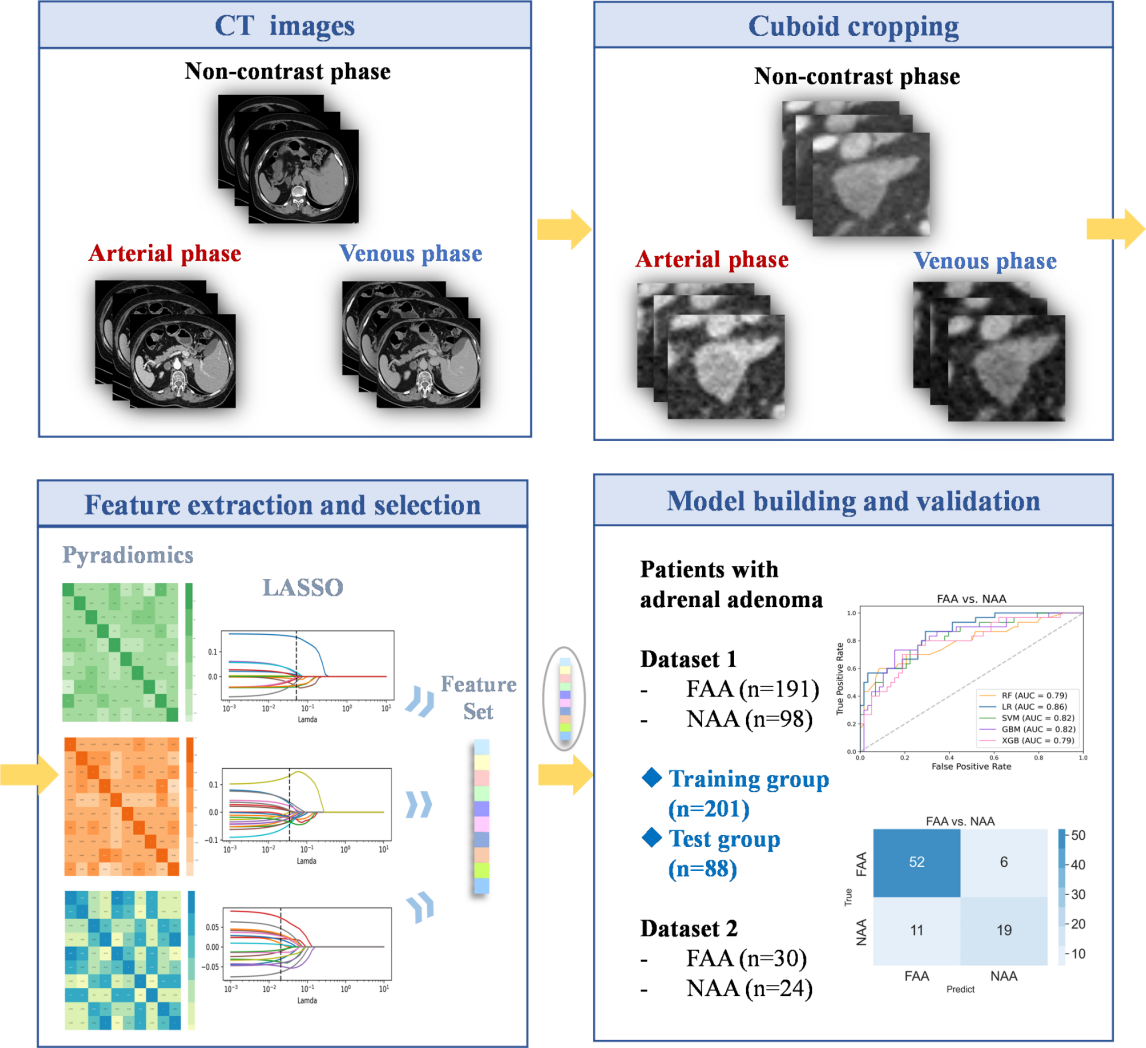
Workflow of the experiment, including CT image acquisition, cuboid cropping, feature extraction and selection, and model building and validation steps

Figure 1 shows the overall workflow of this study. After the acquisition of CT images, the cuboid containing the adrenal adenoma region was cut from the CT images of the arterial, venous, and non-contrast phases. Next, feature extraction and selection were conducted. Finally, a binary classification model was established to determine the final prediction results.

### Cuboid cropping

In the process of cuboid cropping, the central point of the adrenal adenoma was determined first, and then a 64*64*32 cuboid was automatically cropped from the CT image to ensure that the cuboid completely contained the lesion area. In the experiment, the cuboid was cropped from the non-contrast, arterial, and venous phase CT images and used as the region of interest (ROI). One radiologist with 8 years of experience in abdominal imaging cropped the cuboids and another radiologist with 10 years of experience examined and confirmed the cropped results.

### Feature extraction

Pyradiomics software [25] was used to extract features from the cropped cuboids from the three-phase CT images. The extracted features initially included the following seven groups: (a) First Order Features, (b) Shape Features, (c) Gray Level Co-occurrence Matrix (GLCM), (d) Gray Level Size Zone Matrix (GLSZM), (e) Gray Level Run Length Matrix (GLRLM), (f) Neighboring Gray Tone Difference Matrix (NGTDM), and (g) Gray Level Dependence Matrix (GLDM). The definitions and detailed explanations of all the above texture features can be found in the Pyradiomics documentation [26]. Finally, a total of 1,967 radiomic features were extracted from each ROI.

### Feature selection

Feature selection is performed to identify critical and discriminative features and reduce over-fitting risk in the final prediction model [27]. First, an independent two-sample t-test was used to calculate whether each feature was significantly different between the two subtypes of adrenal adenoma. If p < 0.05, the feature was retained. Second, the Least Absolute Shrinkage and Selection Operator (LASSO) was employed to identify the final discriminative features. LASSO improves both prediction accuracy and model interpretability by combining the superior qualities of ridge regression and subset selection [28] and therefore is commonly used for feature selection [29, 30]. LASSO can reduce the coefficient of variables (that have little effect on the regression) to 0 during the fitting process, hence achieving variable screening and complexity adjustment [31, 32].

Only the training set data is used in feature selection. By calculating the coefficients of each variable in the LASSO, the features of the model can be sorted in order of importance from high to low. For every phase of the CT images, the top 10 features were used to train the subsequent two-category classifiers.

The radiomic features from the three phases of CT images were combined to improve the classification performance. First, a two-sample t-test was conducted for features from each phase. Second, the radiomic features that had significant differences were obtained from the three phases of CT images and concatenated into a vector. Third, LASSO regression was used to calculate the coefficients of each feature in the vector. Fourth, the features were sorted and the top 10 features were selected to build the models.

### Machine-learning classification model

Five machine learning algorithms, RF [33, 34], SVM [35, 36], Logistic Regression [37, 38] (LR) Gradient Boosting Machine (GBM), and eXtreme Gradient Boosting (XGBoost), were used to establish two-category classification models. For hyperparameter optimization of the classification model, grid-search was used for 10-fold cross-validation to search for the optimal parameters of the model [39].

The models using single-phase CT features, multi-phase fusion CT features, and radiomic-clinical features were trained, tested, and compared. An independent test set in Dataset 1 was used to evaluate the performance of the model using measures of accuracy (ACC), specificity, sensitivity, and area under the curve (AUC). Receiver operating characteristic (ROC) curves were drawn. Moreover, models with and without the hyperparameter optimization were also compared. Dataset 2 with 54 patients served as the external validation to investigate the model’s generalization capability.

The machine learning algorithms were completed using the open-source Python Scikit-Learn library [40] (Version 0.24.1). All experiments were conducted on Pycharm (Version 2020.2, Python Version 3.7.9). An independent two-sample t-test was used for statistical analysis of the age, gender, and location of adrenal adenomas. A p-value less than 0.05 indicated significant differences between the two independent groups.

## Results

### Demographic and clinical characteristics

The demographic and clinical characteristics of patients with adrenal adenomas in Datasets 1 and 2 are presented in Table 1. In Dataset 1, there was a significant age difference between FAA and NAA patients (p = 0.041). The average age of NAA patients was slightly higher than that of FAA patients, and no significant gender difference was found (p = 0.419). In FAA, 100 patients had lesions on the left, 77 on the right, and 14 on both sides. In NAA, 51, 40, and 7 patients had lesions on the left, right, or both sides, respectively. In Dataset 2, there was no significant difference in gender or age between the two groups.

**Table 1:** Demographic and clinical characteristics

Measure	Dataset 1 (289 patients)			Dataset 2 (54 patients)		
	FAA	NAA	P value*	FAA	NAA	P value*
Number of patients	191	98	—	30	24	—
Sex (F/M)	104/87	59/39	0.419	16/14	13/11	1.0
Age (mean/range) (years)	54.3 (30–76)	57.1 (36–85)	0.041	54.0 (33–74)	56.9 (32–78)	0.382
Lesion side (R/L/B)	77/100/14	40/51/7	—	15/13/2	10/11/3	—

*A two-sample t-test was used for age comparison between the two groups, and the Chi-square test for gender comparison. F, female; M, male; R, right; L, left; B, bilateral.

### Radiomic characteristics

**Fig. 2:**
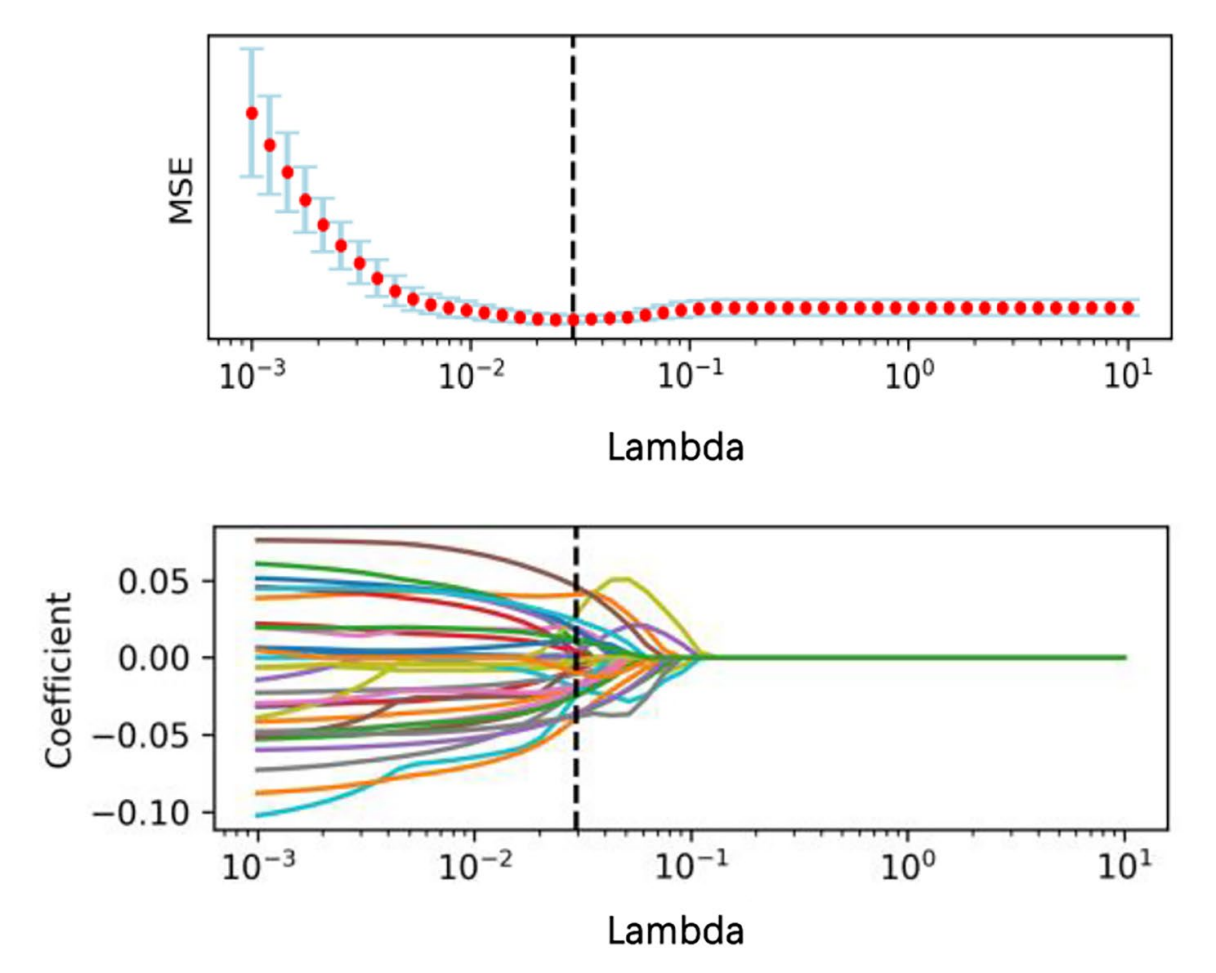
Variation of MSE and coefficient of each feature with Lambda in the LASSO. The features were retained when the three phases were combined (two-sample t-test)

Figure 2 shows the mean square error (MSE) with Lambda in the LASSO and the variation of each feature coefficient with Lambda. A two-sample t-test showed there were 661 features with significant differences, including 146, 343, and 172 from the arterial, venous, and non-contrast phases, respectively. While MSE reached the minimum value marked in the dotted line in Fig. 2, the number of features was reduced to 37.

**Fig. 3:**
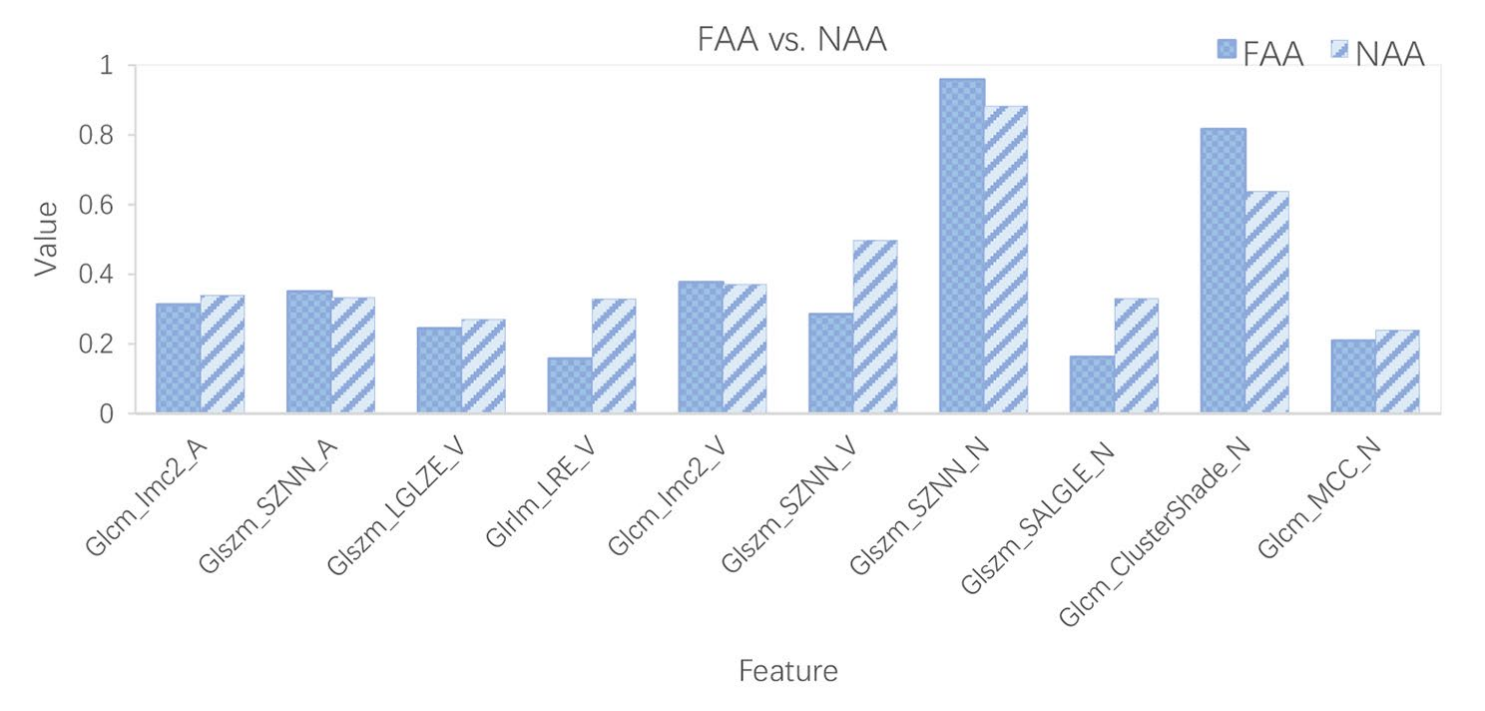
Comparison of features (mean and standard deviation) selected by LASSO from the three CT image phases. It is noteworthy that there is a significant difference between groups, as shown by a two-sample t-test before LASSO.

Using the absolute value of the coefficients, the top 10 discriminate features in a fusion of the three CT image phases are summarized in Table 2. The features (mean and standard deviation) from the CT images of the three phases selected by LASSO are compared in Fig. 3. It is noteworthy that there is a significant difference between groups, as shown by a two-sample t-test before LASSO. In Table 2; Fig. 3, the last letter in the name of the feature indicates which phase the feature is from (A, arterial; V, venous; N, non-contrast).

**Table 2:** Discriminative features in two-category models

No.	Name	Definition	Comparison
1	gradient_glcm_Imc2_A	Distributions of i and j (quantifying the complexity of the texture).	NAA ↑
2	log-sigma-1-mm-3D_glszm_SZNN_A	The measure of the variability of size zone volumes throughout the image.	FAA ↑
3	log-sigma-2-mm-3D_glszm_LGLZE_V	The measure of the distribution of lower gray-level size zones.	NAA ↑
4	wavelet-LHH_glrlm_LRE_V	The measure of the distribution of long run lengths.	NAA ↑
5	wavelet-HLH_glcm_Imc2_V	Distributions of i and j (quantifying the complexity of the texture).	FAA ↑
6	wavelet-HHL_glszm_SZNN_V	The measure of the variability of size zone volumes throughout the image.	NAA ↑
7	exponential_glszm_SZNN_N	The measure of the variability of size zone volumes throughout the image.	FAA ↑
8	log-sigma-2-mm-3D_glszm_SALGLE_N	Distribution of smaller size zones with lower gray levels.	NAA ↑
9	logarithm_glcm_ClusterShade_N	The measure of the skewness and uniformity of the GLCM.	FAA ↑
10	wavelet-LLH_glcm_MCC_N	The Maximal Correlation Coefficient is a measure of the complexity of the texture.	NAA ↑

The mean values of SizeZoneNonUniformityNormalized_A (SZNN), Imc2_V, SZNN_N, and ClusterShade_N were greater in FAA than in NAA. By contrast, NAA had higher mean values for the other features.

### Performance of models using CT features from single phases

**Fig. 4:**
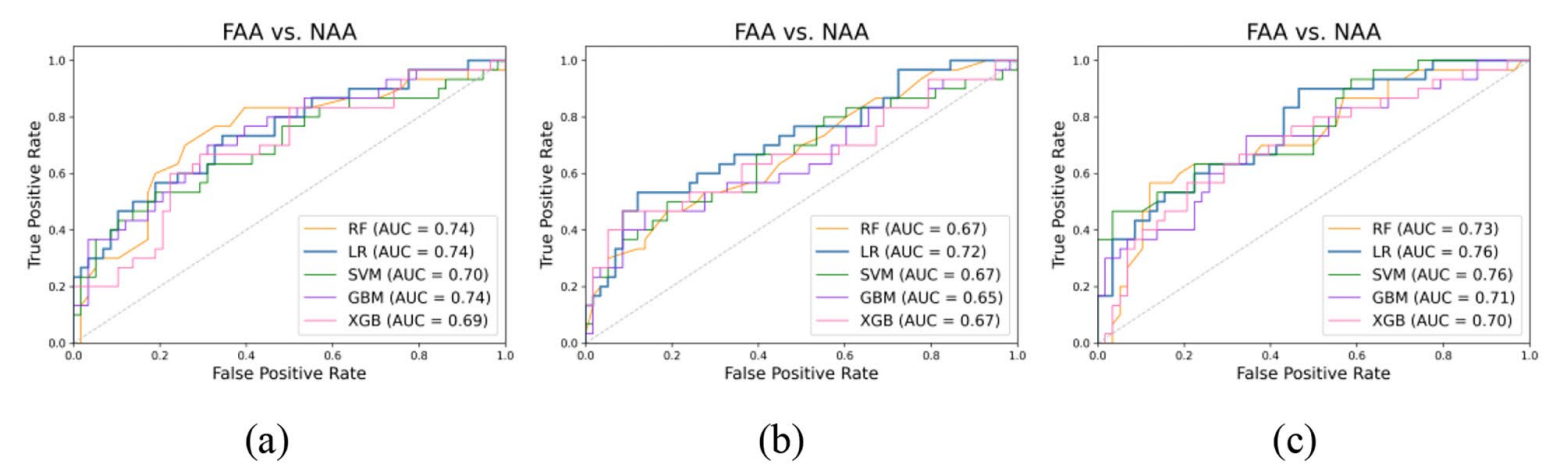
ROC curve of two-category models in each phase. (a) Arterial phase; (b) Venous phase; (c) Non-contrast phase

Figure 4 shows the ROC curve of five classification models with different machine-learning methods for each phase. The AUC of the five models did not change significantly when using features from single phases. Specifically, the AUC range of the five machine learning models was 0.69–0.74 for arterial phase features, 0.65–0.72 for venous phase features, and 0.70–0.76 for non-contrast phase features.

**Table 3:** Prediction performance of five two-category models in each phase

Phase	Classifier	Precision	Recall	Accuracy	AUC
Arterial	RF	65.6%	59.8%	69.3%	0.74
	**LR**	**69.7%**	**66.4%**	**72.7%**	**0.74**
	SVM	72.1%	60.7%	71.6%	0.70
	GBM	68.3%	64.8%	71.6%	0.74
	XGB	61.7%	55.7%	67.0%	0.69
Venous	RF	65.2%	61.4%	69.3%	0.67
	**LR**	**71.2%**	**64.0%**	**72.7%**	**0.72**
	SVM	66.7%	65.5%	70.5%	0.67
	GBM	61.0%	61.2%	64.8%	0.65
	XGB	65.3%	63.9%	69.3%	0.67
Non-contrast	RF	71.2%	64.0%	72.7%	0.73
	**LR**	**76.3%**	**68.2%**	**76.1%**	**0.76**
	SVM	76.3%	68.2%	76.1%	0.76
	GBM	68.1%	66.4%	71.6%	0.71
	XGB	67.4%	61.5%	70.5%	0.70

Bold font indicates the highest accuracy among the five machine learning methods.

Table 3 describes the performance measures of the five machine learning methods using 10 features from the single phases. The highest accuracy was 72.7% in the arterial phase, 72.7% in the venous phase, and 76.1% in the non-contrast phase. In summary, the mean accuracy of the final prediction was less than 80.0% for single phases.

### Performance of models using combined features from all three CT phases

**Fig. 5:**
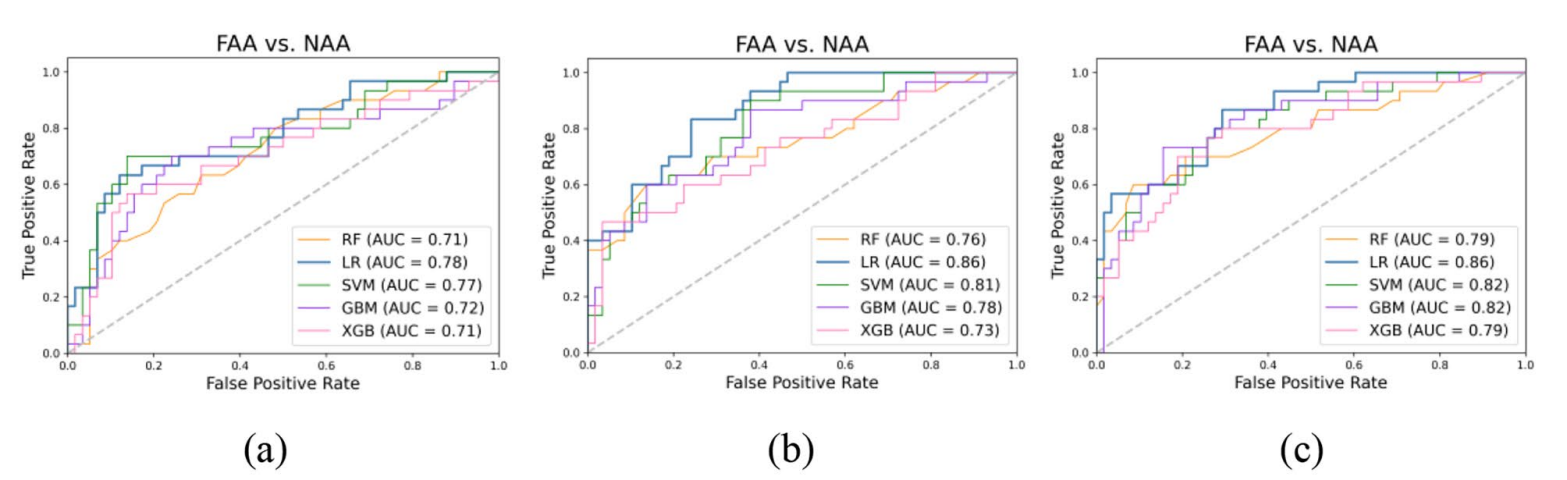
ROC curves of models with combined features from all three CT image phases: (a) no parameter optimization; (b) parameter optimization; (c) clinical features included

The experiment used a grid-search algorithm to optimize the model’s hyperparameters. As a comparison, we first used the models without parameter optimization for prediction analysis. Table 4 shows the performance measures of the five machine learning models without parameter optimization for 10 features in three phases. Figure 5**(a)** shows the ROC curves of the five models. The results show that LR yielded the best performance, with precision, recall, and accuracy of 76.5%, 74.0%, and 78.4%, respectively, and the AUC value was 0.78.

**Table 4:** Prediction performance of five models without parameter optimization using combined features from all three phases in the test set

Classifier	Precision	Recall	Accuracy	AUC
RF	69.0%	63.2%	71.6%	0.71
**LR**	**76.5%**	**74.0%**	**78.4%**	**0.78**
SVM	77.0%	73.2%	78.4%	0.77
GBM	70.0%	65.6%	72.7%	0.72
XGB	70.0%	65.6%	72.7%	0.71

Bold font indicates the highest accuracy among the five machine learning methods.

Table 5 shows the performance measures of five machine learning methods with 10 features in three stages following parameter optimization, and Fig. 5**(b)** shows the ROC curves of these models. Among them, LR performed best, with precision, recall, accuracy, and AUC values of 85.3%, 76.6%, 83.0%, and 0.86, respectively. Parameter optimization improved the accuracy and AUC of the models.

**Table 5:** Prediction performance of five models with parameter optimization using combined features from all three phases in the test set

Classifier	Precision	Recall	Accuracy	AUC
RF	76.5%	70.7%	77.3%	0.76
**LR**	**85.3%**	**76.6%**	**83.0%**	**0.86**
SVM	79.2%	76.2%	80.7%	0.81
GBM	72.2%	72.2%	75.0%	0.78
XGB	70.9%	70.9%	73.9%	0.73

Bold font indicates the highest accuracy among the five machine learning methods.

Meanwhile, the patients had statistically significant age differences, so this clinical information was added to the training feature set to train the models and evaluate the effect of age. Table 6 shows the test results of the models after adding this clinical feature, and Fig. 5**(c)** shows the ROC curve of the five models. The accuracy of the models did not increase significantly after adding the clinical information as a feature, but AUC did increase significantly. Among the models, LR still performed best, with precision, recall, and accuracy values of 85.3%, 76.6%, and 83.0%, respectively, and an AUC of 0.86.

**Table 6:** The prediction performance of five models after adding a clinical feature to the test set

Classifier	Precision	Recall	Accuracy	AUC
RF	76.5%	74.0%	78.4%	0.79
**LR**	**85.3%**	**76.6%**	**83.0%**	**0.86**
SVM	79.7%	73.2%	79.5%	0.82
GBM	72.2%	72.2%	75.0%	0.82
XGB	73.7%	71.4%	76.1%	0.79

Bold font indicates the highest accuracy among the five machine learning methods.

**Fig. 6:**
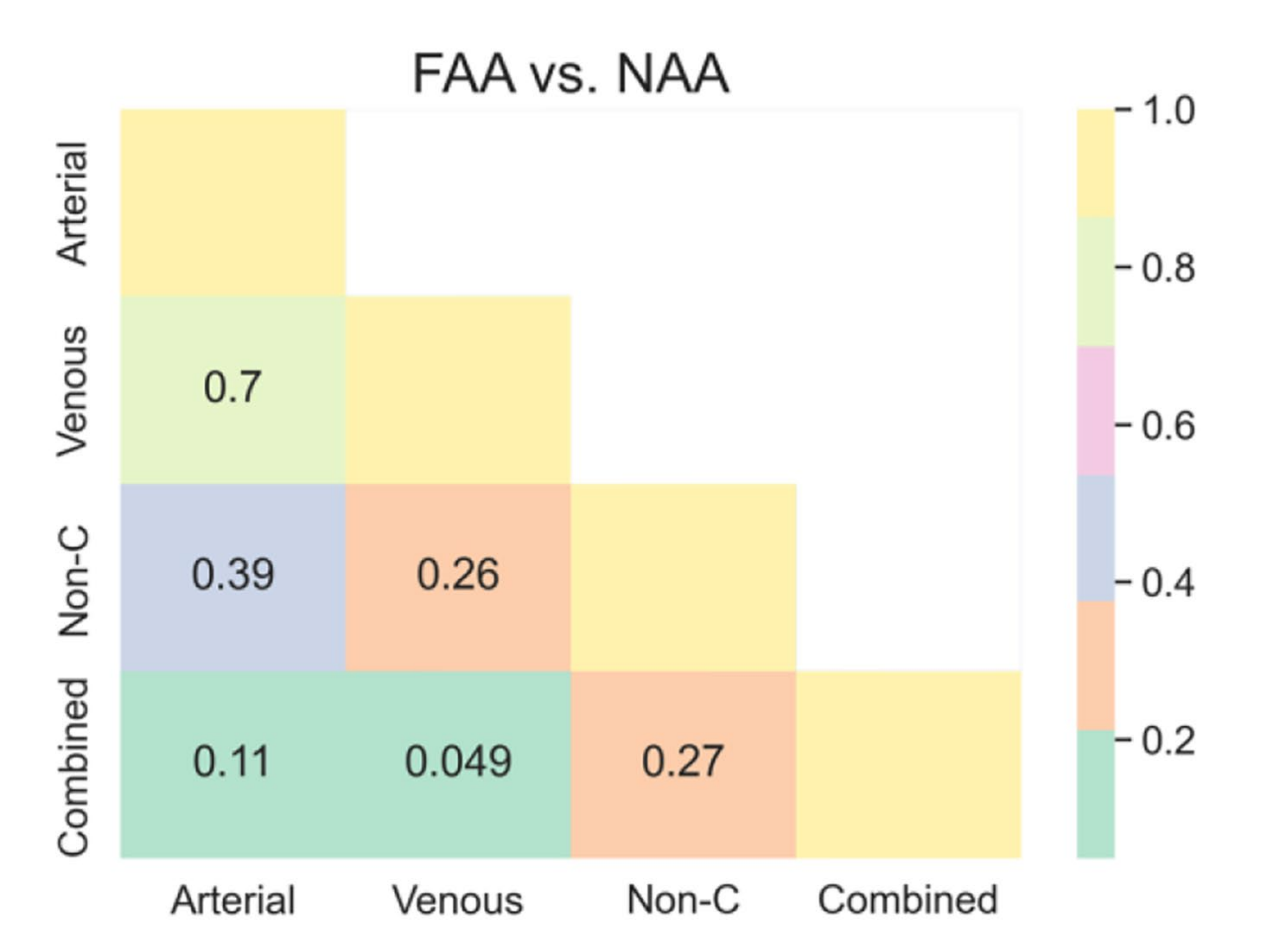
The Delong test compares the ROC curves of the LR model among four CT image sets (three single phases and a combination of the three phases)

The p-value of the Delong test from the ROC curve for the LR models among the four CT image sets (three single phases and a combination of the three phases) is shown in Fig. 6. For FAA vs. NAA, the AUC of the LR model after feature fusion was significantly higher than that of the model using only the venous phase features (Delong test, p = 0.049). Although the AUC was also higher than that of the arterial and non-contrast phase models, no significant differences were observed in the Delong test (p = 0.11, 0.27, respectively).

### Performance using the external validation dataset

**Fig. 7:**
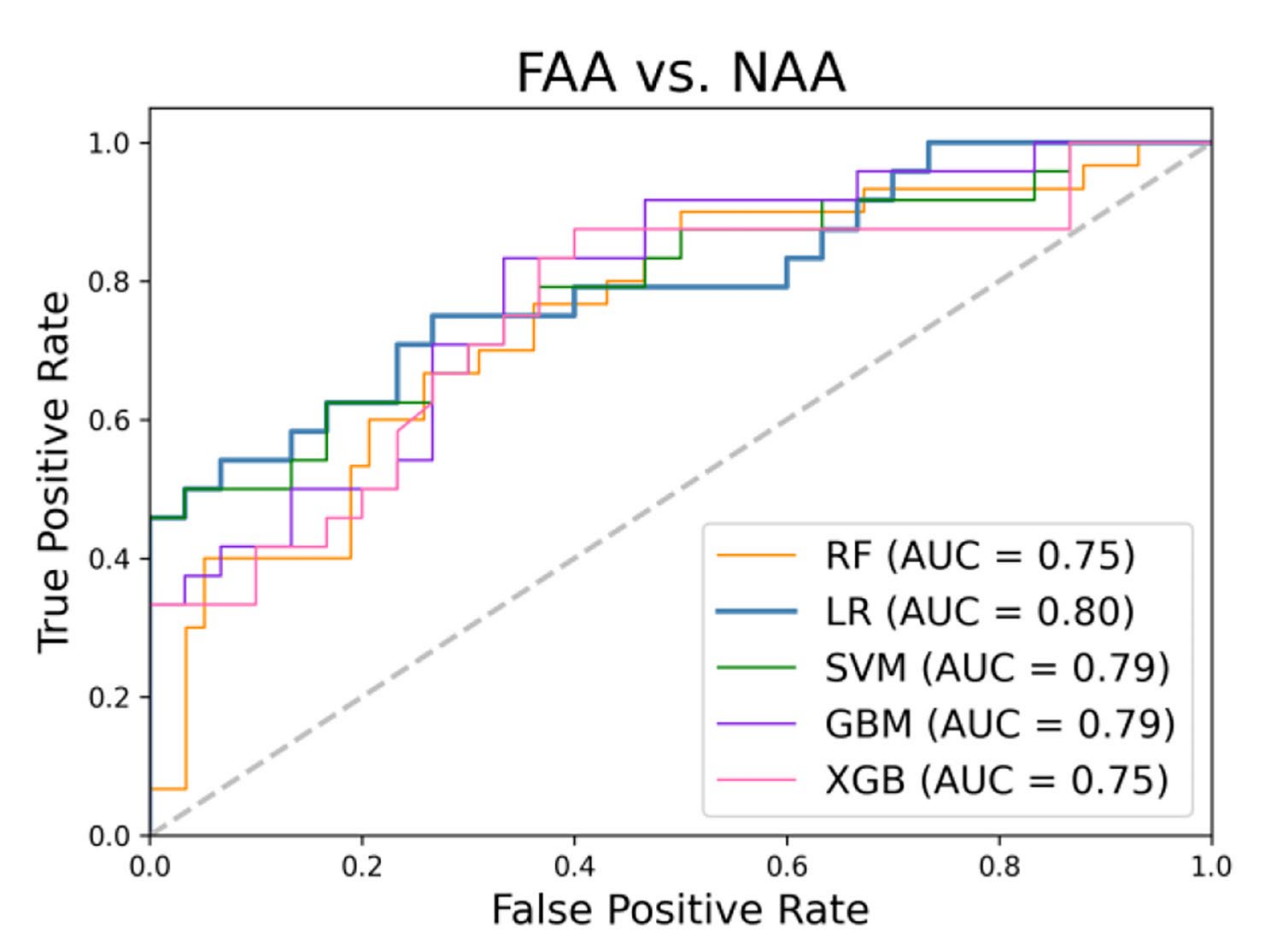
ROC curve of models using the external validation dataset

In the external validation dataset, the performance of the five machine learning models using radiomic-clinical features is presented in Table 7. LR achieved the highest accuracy of 77.8% (42/54). SVM, RF, GBM, and XGBoost yielded accuracies of 77.8% (42/54), 74.1% (40/54), 74.1% (40/54), and 72.2% (39/54), respectively. Figure 7 shows the ROC curve of the five models using the external validation dataset.

**Table 7:** The prediction performance of five models using the external validation dataset

Classifier	Precision	Recall	Accuracy	AUC
RF	84.1%	70.8%	74.1%	0.75
**LR**	**85.7%**	**75.0%**	**77.8%**	**0.80**
SVM	80.6%	75.8%	77.8%	0.79
GBM	75.0%	72.5%	74.1%	0.79
XGB	74.6%	70.0%	72.2%	0.75

Bold font indicates the highest accuracy among the five machine learning methods.

## Discussion

In this study, CT radiomic features extracted from CT images have been utilized to differentiate between functioning and non-functioning adrenal adenomas using machine learning models. Differences in demographic and clinical characteristics were observed between the two groups. Ten discriminative features from three-phase CT images were identified and analyzed while differentiating between the two subtypes. The accuracy of the final prediction using LR can reach as high as 83.0%.

### Clinical significance of differentiating between adrenal adenoma subtypes

Adrenal adenomas are common urinary tract tumors. For tumor-based diseases, patients and their families are concerned about effective treatment, and early diagnosis of the disease is of great importance for determining the proper method and timing of subsequent treatments [41].

FAA and NAA require different follow-up treatments, including the type of medication and the need for surgery with its attendant risks. There are several difficulties with the current methods of clinical diagnosis. Radiologists cannot always make an accurate diagnosis directly from CT images, which can lead to unnecessary treatment [10]. Currently, patients with FAA need to have multiple invasive blood tests, often including adrenal venous sampling, to measure hormone levels that serve to guide surgeons in resecting the functioning lesion(s). Therefore, advanced diagnosis of NAA without invasive blood tests could reduce risk and discomfort for patients while simplifying and accelerating testing and treatment.

Using our models described here, the accuracy of differentiating FAA from NAA was more than 80%. This method can help doctors quickly estimate the type of adrenal adenoma, rapidly determine the corresponding effective treatment plan, and minimize unnecessary lab testing of patients who do not present with hormonal imbalance. In addition, this method is completely non-invasive, simple, and fast. The only material required is the patient’s CT images, which are routinely collected in the clinic.

### Discriminative features between the two subtypes

Most of our patients with FAA and NAA were female, which is consistent with the results of previous studies showing that the incidence of adrenal adenomas is higher in women [5, 42]. In terms of age, FAA typically develops earlier in life than NAA, while the average age is over 50 years, demonstrating that the incidence of adrenal adenomas increases with age [2, 43, 44].

Cluster Shade is a quantity that describes the skewness and uniformity of the GLCM, with higher values indicating greater asymmetry about the mean. The value of Cluster Shade in assessing FAA is higher than that for NAA, which indicates NAA has a more uniform gray level distribution in CT images. Because of the differences in tumor cells that make up FAA, tumor cell morphology may be heterogeneous, thus increasing the heterogeneity of FAA in histopathology [45, 46].

SZNN is a measure of the variability of size zone volumes throughout the image, with a lower value indicating more homogeneity among zone size volumes in the image. The SZNN feature value for FAA was greater than for NAA, and therefore NAA was more homogeneous. The etiology of FAA includes extensive adrenal hyperplasia, which may show clinical, morphological, and molecular heterogeneity [45]. Long Run Emphasis (LRE) is a measure of the distribution of long run lengths, with a greater value indicative of long run lengths and more coarse structural textures. [25]. LRE is higher for NAA than FAA, so NAA has a rougher texture. This may correlate with tumor micro-environment heterogeneity [47]. Therefore, these key radiomic features can assist in analyzing the texture characteristics of tumors and evaluating the symmetry and heterogeneity of tumors.

### Model performance after feature fusion

From the experimental results, we have found that among the classification models trained after the fusion of equivalent features from the three phases, LR had the best performance, with an accuracy of 83.0%. Compared with the LR models that only used a single-phase feature, performance improved significantly (arterial phase: 72.7%, venous phase: 72.7%, and non-contrast phase: 76.1%). The Delong test [48] confirmed this observation. Therefore, regardless of the accuracy or AUC of the model, fusing features from the three CT image phases to train the model is better than using only features from a single phase.

### Methodological advantages

Table 8 compares our methods with those in previous studies, where three methodological advantages are apparent. First, in our study, we cropped three-dimensional 64*64*32 cuboids containing entire lesions, and features were extracted from 3D ROIs in CT images. Our method contains more feature information than previous studies using features extracted from a single 2D representative section of the largest tumor [49]. Daye et al. [24] used SVM to predict the prognosis of patients with metastatic adrenal tumors. However, the ROI was delineated only on the largest section of the lesion, while the characteristics of the entire 3D tumor were not considered.

Second, more information about the tumor was obtained in our study by fusing features from non-contrast-, arterial- and venous-phase CT images. Koo et al. [50] analyzed the value of 15-minute-delayed contrast-enhanced CT and chemical shift magnetic resonance (CSMR) for identifying adrenal masses and discovered that the delay-enhanced images were more effective for diagnosing adrenal sebaceous adenoma. In the study of Feng et al. [19], feature analysis and selection were performed in three phases of CT images, and the optimal feature subset was finally selected for the construction of the machine learning classification model.

Third, the model we developed can diagnose both FAA and NAA. At the same time, we collected patients’ CT data from two hospitals for validation. Previous studies have primarily focused on the distinction between benign and malignant adrenal adenomas [50, 51], while few studies have distinguished between functioning and non-functioning adrenal adenomas [52]. Additionally, there is no relevant research on the automatic differentiation of adrenal adenomas based on CT images.

**Table 8:** Comparison of our methods to those of previous studies

Study	Key aspects	Performance
Our Method	− 343 CT scans of patients (two datasets), 221 in FAA, 122 in NAA - Feature fusion of three phases - Feature selected by T-test and LASSO - Binary classification (FAA or NAA)	Accuracy = 0.830 Specificity = 0.853 Sensitivity = 0.766 AUC = 0.86
Elmohr et al., 2019 [22]	− 54 CT scans of patients - Analysis of the textural features and the attenuation values - Univariate logistic regression and Boruta random forest - Binary classification (large adrenal adenomas or carcinomas)	Accuracy = 0.820 Specificity = 0.830 Sensitivity = 0.810 AUC = 0.89
Moawad et al., 2021 [23]	− 40 CT scans of patients, 21 in benign, 19 in malignant - Select the final discriminative features using RFE - Binary classification (benign or malignant)	Accuracy = 0.775 Specificity = 0.714 Sensitivity = 0.842 AUC = 0.85
Koo et al., 2013 [50]	− 453 patients examined with 15-DECT and 217 patients examined with CSMR - Delayed contrast-enhanced CT and chemical shift magnetic resonance characterize adrenal lesions - Binary classification (adenoma and non-adenoma)	Accuracy = 0.881 Specificity = 0.748 Sensitivity = 0.917
Vos et al., 2020 [51]	− 96 patients, 74 in malignant, 22 in benign - ^18^ F-FDG‐PET SUVmax cutoff value of ≥ 4.6 - Binary classification (benign or malignant)	Specificity = 0.830 Sensitivity = 0.760 AUC = 0.857
Chen et al., 2021 [52]	− 125 serum samples, 84 patients with adrenal tumor - SERS peak analysis - PCA-SVM was applied on serum SERS measurements - Binary classification (FAA or NAA)	Accuracy = 0.845 Specificity = 0.886 Sensitivity = 0.800 AUC = 0.899

### Limitations and future work

This study has a few limitations. First, although the number of cases of each subtype was larger than in previous studies, the sample size was relatively small, with about 100 NAA patients. Second, all patients were retrospectively registered from hospitals in the same region, and therefore the generalizability of the model is unclear. In the future, more data from multiple hospitals in multiple regions needs to be collected for testing and verification. Third, the final LR accuracy of the three-phase classification is 83.0%. Although it is a satisfactory result, the prediction would have been in error for nearly one-fifth of the patients with adrenal adenoma. We expect more high-quality CT images from different hospitals to be collected shortly, and advanced deep-learning methods may further improve the model’s predictive power. Additionally, more clinical non-image information can be used to optimize the model.

## Conclusion

The radiomic features from the lesion region in three-phase CT images can potentially suggest the functioning or non-functioning nature of adrenal adenoma. The discriminative features identified here may help in understanding the heterogeneity of adrenal adenoma. The resulting radiomic models can be a non-invasive, low-cost, and rapid method to reduce unnecessary testing in asymptomatic patients with incidentally discovered adrenal adenoma.

## Data Availability

The datasets used and/or analysed during the current study are available from the corresponding author on reasonable request.

## List of abbreviations

AUC: Area under the curve
CS: Cushing’s syndrome
CT: Computed tomography
GLCM: Gray level co-occurrence matrix
GLDM: Gray level dependence matrix
GLSZM: Gray level size zone matrix
GLRLM: Gray level run length matrix
LASSO: Least absolute shrinkage and selection operator
ML: Machine learning
MSE: Mean square error
FAA: Functioning adrenocortical adenoma
NAA: Non-functioning adrenocortical adenoma
NGTDM: Neighboring gray tone difference matrix
PHA: Primary hyperaldosteronism
RF: Random Forest
ROC: Receiver operating characteristic
SVM: Support vector machine
GBM: Gradient Boosting Machine
XGB: eXtreme Gradient Boosting
